# Association of elevated Delta-like canonical Notch ligand 1 levels with clinical outcomes in patients hospitalized for SARS-CoV2 infection

**DOI:** 10.1038/s41598-025-03673-6

**Published:** 2025-05-27

**Authors:** Jan Niklas Thon, Judith Schenz, Uta Merle, Maximilian Dietrich, Vivienne Theobald, Markus Alexander Weigand, Benedikt Hermann Siegler

**Affiliations:** 1 Medical Faculty Heidelberg, Department of Anesthesiology, Heidelberg University, Im Neuenheimer Feld 420, 69120 Heidelberg, Baden-Württemberg Germany; 2 Medical Faculty Heidelberg, Department of Gastroenterology and Infectious Diseases, Heidelberg University, Im Neuenheimer Feld 420, 69120 Heidelberg, Baden-Württemberg Germany; 3 Medical Faculty Heidelberg, University Center for ARDS and Weaning, Heidelberg University, Röntgenstraße 1, 69126 Heidelberg, Baden-Württemberg Germany

**Keywords:** COVID-19, sDLL1, Notch signaling pathway, Viral sepsis, Biomarkers, Viral infection

## Abstract

Soluble Delta-like ligand 1 (sDLL1) has demonstrated promising results as an early biomarker of bacterial sepsis, but its role in viral infections remains unclear. This study investigated the association between sDLL1 levels and clinical outcomes in patients hospitalized for COVID-19. In this secondary analysis of a single-center prospective observational trial, we measured plasma sDLL1 levels in 46 patients admitted with PCR-confirmed SARS-CoV2 infection between December 2020 and April 2021. Patients were divided into a high-sDLL1 group, upper quartile of patients with the highest measured sDLL1 levels, and low-sDLL1 group, lower three quartiles of patients. Clinical outcomes, including secondary infections, organ dysfunction, and mortality, were compared between groups. Patients in the high-sDLL1 group (*n* = 11, 24%) showed higher rates of secondary infections (63% vs. 20%, OR 7, CI 1.6 to 31, *p* = 0.01) with higher odds of pulmonary secondary infections (46% vs. 11%, OR 6.5, CI 1.3 to 31, *p* = 0.03). Organ dysfunction was more prevalent in the high-sDLL1-group, indicated by a higher maximal Sequential Organ-Failure Assessment (SOFA) score (median (IQR) 11 (8.5–14) vs. 3 (0.5-8), *p* < 0.01) as well as higher rates of vasopressor support (64% vs. 26%, OR 5.1, CI 1.2 to 21, *p* = 0.03) and renal replacement therapy (36% vs. 9%, OR 6.1, CI 1.1 to 3.6, *p* < 0.05). The high-sDLL1 group also showed increased 90-day mortality (45% vs. 11%, OR 6.5, CI 1.3 to 31, *p* = 0.03). These findings suggest that high levels of sDLL1 are associated with adverse outcomes in viral sepsis, warranting further investigation in larger, prospective studies.

**Supplementary Information:**

The online version contains supplementary material available at 10.1038/s41598-025-03673-6.

## Introduction

The global pandemic set off by the severe acute respiratory syndrome coronavirus 2 (SARS-CoV2) has accounted for high rates of hospitalization and mortality for more than two years^1–3^. During that period the understanding between the clinical presentation of COVID-19, symptoms ranging from dyspnea to acute respiratory distress syndrome (ARDS), and the underlying immune response upon infection has progressed^4^. Recent publications suggest the Notch system as a bridge between the innate and adaptive immune system and as a potential therapeutic target to alter disease severity^5–7^.

In mammals, the Notch signaling network is made up of four Notch receptors (Notch1-4) and five Notch receptor ligands (Jagged 1 and 2 and delta like canonical Notch ligand (DLL) 1, 2, and 3)^8,9^. Notch receptors are transmembrane proteins consisting of one intracellular and one extracellular domain. Upon ligand binding a series of proteolytic events leads to the cleavage of both receptor domains. The Notch intracellular domain (NICD) enters the nucleus and binds to the DNA-binding protein CBF1 and Mastermind-like transcriptional coactivator 1 (MAML1) to stimulate gene transcription^9^. Subsequently the extracellular domain, consisting of receptor and ligand, is measurable in plasma (i.e. soluble DDL1 (sDLL1) in case of DLL1 binding)^10^. In vitro experiments showed an increase in DLL1 expression on monocytes and IL-6 secretion after LPS-stimulation, which led to an activation of the transcription factor STAT3 and a subsequent upregulation of DLL1 and programmed death-ligand 1 (PD-L1) on monocytes^10^. Overexpression of PD-L1 on monocytes may lead to both, a reduction in T-cell proliferation with less activated CD4 + T-cells and more T regulatory cells (Treg), as well as an induction of the development of immune tolerant antigen presenting cells.

In critically ill patients with sepsis, Hildebrand et al. were able to demonstrate a better performance of sDLL1 as a diagnostic biomarker compared to conventional biomarkers for inflammation like C-reactive protein (CRP), procalcitonin (PCT), and white blood cell count (WBC)^11^.

In a study including patients undergoing liver transplantation, sDLL1 further showed promising results as a biomarker for early detection of secondary infections^12^. In case of viral infections, Ito et al. found increased expression of DLL1 on macrophages during influenza virus H1 N1 infection. By blocking Notch signaling through antibodies against DLL1 or γ-secretase inhibitor, thus inhibiting the cleavage of the intracellular domain of the Notch receptor, mortality, inflammation, and virus load increased while IFN-γ levels were reduced^13^. Additionally, DLL1 expression on macrophages impacted INF-γ secretion by CD4 + and CD8 + T-cells, demonstrating the importance of Notch signaling in the body’s anti-viral immune response^8,13^. Both diagnostic and prognostic properties of sDLL1 during critical illness were evaluated by Hölle et al. in different cohorts with inflammatory diseases, infection, and sepsis. By using cut-off values between 10,623 pg/ml and 13,830 pg/ml sDLL1 showed a better diagnostic performance to discriminate between septic and non-septic patients and was found superior for prediction of 28-day mortality compared to CRP, PCT, WBC^14^.

Despite the above-mentioned research highlighting the potential of sDLL1 as both diagnostic and prognostic biomarker in critically ill patients, there is still a paucity of data on an association of sDLL1 levels with clinical outcomes. In this study, we measured plasma sDLL1 levels and performed a retrospective analysis of data collected as part of a prospective observational study including patients hospitalized due to PCR-confirmed SARS-CoV2 infection. Based on previously published cut off values ranging up to 30,000 pg/ml^11^, we hypothesized that patients with the highest sDLL1 levels in our cohort might have a higher mortality, increased rates of secondary infections and a higher prevalence of organ failure.

## Results

### Study cohort and patients’ characteristics

**Fig. 1:**
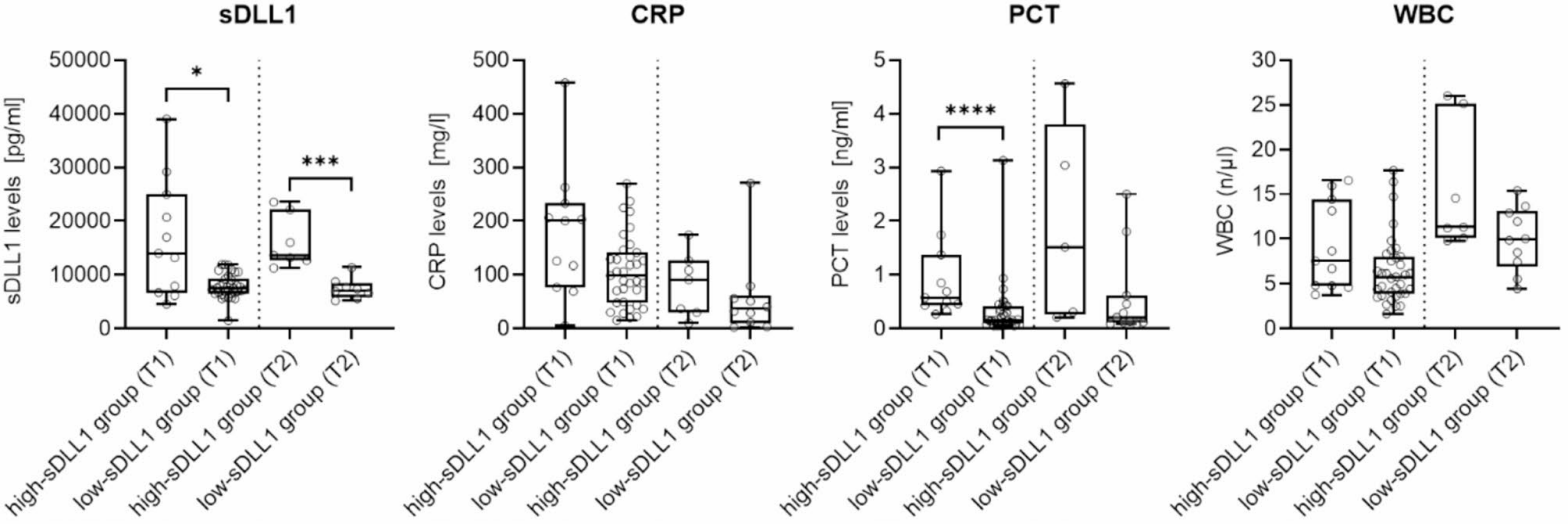
Levels of sDLL1 and routine clinical inflammatory biomarkers. sDLL1, white blood cell count (WBC), procalcitonin (PCT) and C-reactive protein (CRP) were measured on day 1 (T1) and day 7 (T2) after study inclusion. Data are presented as box and whiskers (min to max). Group comparisons between the high-sDLL1-group (*n* = 11 patients at T1, *n* = 7 at T2) and the low-sDLL1-group (*n* = 35 patients at T1, *n* = 10 at T2) were performed by two-sided Mann-Whitney U-test, with `****´ indicating p-values < 0.0001, `***´ indicating p-values *p* < 0.001 and `*´ indicating p-values < 0.05. For *n* = 2 patients, no PCT-values were available at T2.

Overall, 46 patients with a median age of 62 years and a male-to-female ratio of 3.2 to 1 were analyzed. Blood samples were available for all patients within 24 h after hospitalization (T1) and for 17 individuals after seven days (T2), as illustrated in supplemental Fig. 1. Based on measured sDLL1-levels, the cohort was divided by quartile, including 35 individuals (76%) in the low-sDLL1-group (representing the three lower quartiles) and 11 individuals (24%) in the high-sDLL1-group (representing the upper most quartile). Median sDLL1-levels were 13,991 pg/ml (IQR 6,713 to 24,981) in the high-sDLL1 group compared to 7,550 pg/ml (IQR 6,565 to 9,316) in the low-sDLL1 group at T1 (*p* = 0.02) and 13,608 (IQR 12,640 to 22,103) vs. 7,550 pg/ml (IQR 5,164 to 7,983, *p* = 0.0001) at T2, respectively. Figure 1 illustrates levels of sDLL1 and routine clinical inflammatory biomarkers at the different time points.

Baseline socio-demographic and clinical characteristics of all patients are listed in Table 1. In total, 27 individuals were admitted to ICU at the time of study inclusion, comprising 9 patients (82%) in the high-sDLL1-group and 18 patients (51%) in the low-sDLL1-group (odds ratio (OR) 4.3, 95% confidence interval (CI) 0.8 to 23, *p* = 0.09).

**Table 1:** Demographics and clinical characteristics. Abbreviations: BMI, body mass index; CCI, Charlson comorbidity index; ICU, intensive care unit.

Variables	Total (*n* = 46)	high-sDLL1 group (*n* = 11)	low-sDLL1 group (*n* = 35)
Age, median (range)	62 (24–95)	70 (48–95)	61 (24–91)
Male sex, n (%)	35 (76)	9 (82)	26 (74)
BMI, median (range)	28 (17–45) ^1^	29 (22–45)	28 (17–40)
Comorbidities			
Cardiovascular, n (%)	11	5 (46)	6 (17)
Respiratory, n (%)	8	2 (18)	6 (17)
Renal, n (%)	4	4 (36)	0 (0)
Cerebral, n (%)	3	1 (9)	2 (6)
Diabetes mellitus, n (%)	14	3 (27)	11 (31)
CCI, mean (range)	1.4 (0–10)	2.6 (0–10)	1 (0–4)
Admission to ICU, n (%)	27 (59)	9 (82)	18 (51)

^1^ Body weight and length to calculate the BMI were available for *n* = 45 patients.

### Association between sDLL1-levels and clinical outcomes

Table 2 summarizes associations between sDLL1-levels and clinical outcomes. During their hospital stay, patients in the high-sDLL1-group were more likely to suffer from secondary infections (63% vs. 20%, OR 7.0, CI 1.6 to 31, *p* = 0.01) with higher odds of pulmonary secondary infections (46% vs. 11%, OR 6.5, CI 1.3 to 31, *p* = 0.03) compared to the low-sDLL1-group.

**Table 2:** Clinical outcomes. Abbreviations: ICU, intensive care unit; RRT, renal replacement therapy.

Variables	Total (*n* = 46)	high-sDLL1 group (*n* = 11)	low-sDLL1 group (*n* = 35)	*p*-value
Secondary infections, total n (%)	14 (30)	7 (63)	7 (20)	0.01
Pulmonary, n (%)	9 (20)	5 (46)	4 (11)	0.03
Urinary, n (%)	1 (2)	0 (0)	1 (3)	1.0
Catheter, n (%)	2 (4)	1 (9)	1 (3)	0.43
Blood stream (%)	2 (4)	1 (9)	1 (9)	0.43
Acute pulmonary failure, n (%)	20 (44)	7 (64)	13 (37)	0.12
Duration of mechanical ventilation, median days (IQR)	0 (0–14)	7 (0–23)	0 (0–9)	0.15
Nosocomial pneumonia per 1000 ICU days, n	31	40	24	0.89
Cardiovascular failure, n (%)	16 (35)	7 (64)	9 (26)	0.03
Duration of vasopressor therapy, median days (IQR)	0 (0–8)	6 (0–24)	0 (0–1)	0.05
Need for RRT, n (%)	7 (15)	4 (36)	3 (9)	< 0.05
Duration of RRT, median days (IQR)	0 (0–0)	0 (0–7)	0 (0–0)	0.16

In addition, organ dysfunction was more prevalent in the high-sDLL1-group, indicated by a higher maximal Sequential Organ-Failure Assessment (SOFA) score (median (IQR) 11 (8.5 to 14) vs. 3,0 (0.5 to 8.0), *p* < 0.01). Patients in the high-sDLL1-group were characterized by higher rates of cardiovascular failure requiring vasopressor support (64% vs. 26%, OR 5.1, CI 1.2 to 21, *p* = 0.03) with a median duration of 6 days of vasopressor therapy as well as higher odds of RRT requirement (36% vs. 9%, OR 6.1, CI 1.1 to 3.6, *p* < 0.05).

### Length of stay and mortality

Length of stay and mortality are reported in Table 3. With a mean of 27 days (IQR 14 to 31 days), patients in the high-sDLL1-group tended to have a longer hospital stay compared to those in the low-sDLL1-group (mean 18 days, IQR 5 to 19 days), yet this difference did not reach statistical significance (*p* = 0.06). Higher 90-day mortality rates were observed in the high-sDLL1-group (45% vs. 11%, OR 6.5, CI 1.3 to 31, *p* = 0.03).

**Table 3:** Length of stay and Mortality. Abbreviations: ICU, intensive care unit; SD, standard deviation.

Variables	Total (*n* = 46)	high-sDLL1 group (*n* = 11)	low-sDLL1 group (*n* = 35)	*p*-value
Length of ICU stay, mean (SD)	13 (20)	18 (20)	11 (20)	0.11
Length of hospital stay, mean (SD)	20 (20)	27 (20)	18 (20)	0.06
Mortality at 30-day, n (%)	7 (15)	3 (27)	4 (11)	0.21
Mortality at 90-day, n (%)	9 (20)	5 (45)	4 (11)	0.03

## Discussion

In times of the SARS-CoV2 pandemic and the ensuing years critical care physicians have been struggling to identify reliable biomarkers for viral sepsis to guide clinical practice. Soluble DLL1 shows promising potential in bacterial sepsis, however, there has been limited research concerning its role in viral infections and viral sepsis^11^. Hildebrand et al. showed a correlation between elevated plasma levels of sDLL1 and severe disease progression in patients with dengue virus^15^. Here we measured sDLL1 plasma levels in 46 hospitalized patients admitted to the ICU and normal ward with a SARS-CoV-19 infection to investigate if elevated sDLL1 levels were associated with worse clinical outcomes. Patients with sDLL1 serum levels in the upper quartile had significantly more secondary infections, displayed higher rates of renal and cardiac failure and showed an increased 90-day mortality. These findings are consistent with previous research focusing on bacterial sepsis^11,12,16^. To our knowledge, this study is the first to show an association of high sDLL1 and adverse clinical outcomes in COVID-19.

In recent years sDLL1 has been shown to be a biomarker of interest when it comes to early detection of bacterial infection in sepsis and in patients undergoing surgical procedures^11,12,14^. Decker et al. could show an early elevation of sDLL1 plasma levels in patients following liver transplantation who suffered from a secondary bacterial infection^12^. Hildebrandt et al. had similar results in sepsis patients and were able to show an early rise and a continues high plasma concentration in sDLL1 levels in patients with bacterial sepsis compared to patients after surgery or suffering from trauma^11^. In our collective there was also an association between elevated sDLL1 plasma levels and secondary infections. This confirms the importance of future work to establish sDLL1 as a possible diagnostic marker for infectious complications.

In this line sDLL1 could also be used as a biomarker to predict mortality or complications during hospitalization. Decker et al. could show that patients who had continuously elevated sDLL1 plasma levels two days after receiving a liver transplant had a complicated hospital stay with higher rates of acute rejection or graft failure and increased rates of organ failure or death^12^. Albeit there were several confounders that might contribute to an accumulation of sDLL1, including the use of immunosuppressive medication. Accordingly, Hölle et al. were able to show an association of sDLL1 elevation with 28-day mortality in a septic collective^14^.

Besides an association between sDLL1 plasma levels and the occurrence of secondary infections, we found higher needs for vasopressor therapy in the high-sDLL1 group of our study. It is well known that sDLL1 and Notch signaling in general play an important role in the formation of chronic heart failure. Norum et al. were able to show a correlation between sDLL1 plasma levels and diastolic dysfunction in patients with DCM and chronic heart failure^17,18^. They could also demonstrate these results in patients with no impaired kidney function, suggesting that an increase in sDLL1 plasma levels does not solely indicate impaired renal function^18^. However, so far, there is little knowledge about sDLL1 in the context of acute heart failure or cardiovascular failure. In bacterial sepsis the impaired endothelial cell function and the subsequent loss of vasoregulation and barrier function are main drivers for increased use of vasopressors to maintain cardiovascular hemostasis and to prevent further organ damage^19,20^. Moll et al. could also demonstrate a direct correlation between DLL1 Notch signaling and lipopolysaccharide (LPS)-mediated endothelial dysfunction leading to eventual vascular leakage in bacterial sepsis^21^. In our study the higher need for vasopressors in the high-DLL1 cohort suggests there might be similarities between viral and bacterial sepsis when it comes to loss of endothelial cell function and cardiovascular failure.

There are several studies investigating the association between sDLL1 plasma levels and renal function. In our study, we found a higher need for RRT in the high-sDLL1 group. Of note, we also found a higher rate of preexisting renal dysfunction in this group. So far, the interplay between renal function and a possible accumulation of sDLL1 remains still unclear. However, three out of four patients in the high sDLL1 group required de novo RRT, indicating a possible use of sDLL1 as a biomarker to access renal function in critically ill patients.

Although the results of our study provide valuable insights, there are limitations including those inherent in the study’s design and the statistical approach that require careful consideration. First, our study was conducted as a secondary analysis of data obtained within a prospective single center trial including a limited sample size. Consequently, the presented findings should be considered exploratory and hypothesis-generating, warranting confirmation in larger, prospective cohorts. Even though our data provide new and valuable information including the association of elevated sDLL1 levels with adverse clinical outcomes in patients hospitalized for SARS-CoV2 infection, we can not rule out that high sDLL1 levels may reflect general illness severity rather than susceptibility to secondary infections. In line, we recently found sDLL1 to be a better predictor of ICU- and in-hospital mortality in patients with sepsis and septic shock than traditional biomarkers such as PCT or lactate. Similar to the results presented here, patients with higher sDLL1 levels had higher degrees of organ dysfunction indicated by a higher need of organ support^22^. Second, to assess potential associations with clinical outcomes, quartile-based stratification of sDLL1 plasma levels was chosen in the present study. This stratification method enables unbiased exploration across the complete range of observed sDLL1 plasma values and avoids any expectations regarding biomarker distribution. While the identification of an optimal cut-off (i.e. via the Youden Index) may provide additional insights, we refrained from defining such a definite threshold to minimize the risk of optimism bias and overfitting. Future research in independent cohorts – ideally with serial sDLL1 measurements and protocol-based microbial testing – will be needed to identify and validate potential sDLL1 thresholds with prognostic utility in the setting of viral infections. Third, the study period coincided with the transition from wild-type to Alpha variants of SARS-CoV-2. As a result, the findings of the present study may have limited generalizability to infections caused by later SARS-CoV-2 variants or to other viral pathogens. Additionally, no SARS-CoV-2 vaccines were available during the study period, which may further limit the applicability of the results to vaccinated populations. Furthermore, the heterogeneity observed among patients admitted to the ICU could have an impact on the interpretation of results, thus explaining why the findings regarding admission rates and the length of stay at an ICU are not statistically significant. Finally, occult bacterial or fungal co-infections at the time of study inclusion may have also had an impact on sDLL1 plasma levels, which we are not able to rule out for certain. Considering these limitations, it is necessary to conduct further prospective studies to determine the utility of sDLL1 is as a biomarker for viral sepsis.

In summary, we were able to show that elevated sDLL1 levels in patients hospitalized due to COVID-19 were associated with the risk of renal replacement therapy, the need for vasopressors, a higher rate of secondary infections as well as a higher 90-day mortality. Whether sDLL1 could serve as a biomarker for adverse events in patients with viral infections should be further addressed in prospective studies in both COVID-19- and non-COVID-19 populations.

## Conclusions

Soluble Delta-like ligand 1 might be a promising biomarker in COVID-19 patients, with elevated levels associated with increased rates of secondary infections, organ dysfunction, and higher 90-day mortality. Larger, prospective studies are warranted to confirm the role of sDLL1 in viral infections and its utility in clinical risk stratification.

## Methods

### Study cohort and ethics

This is a secondary analysis evaluating data and samples of a prospectively enrolled study cohort including patients hospitalized at Heidelberg University Hospital between December 29, 2020, and April 27, 2021 due to PCR-confirmed viral infection with SARS-CoV2^23^. Participants had to be at least 18 years of age and had to be admitted to the hospital due to COVID-19 no longer than 24 h before study inclusion. General exclusion criteria were enrolment in an interventional study, pre-existing immunosuppression, chronic viral infections (e.g. HIV, HCV, HBC), pregnancy, or anemia. In addition, patients with a coincidence of SARS-CoV2 infection and other acute infections at the time of admission (i.e. patients with abdominal sepsis) were not included.

The study was conducted after approval by the local ethics committee of the medical faculty of Heidelberg University (Alte Glockengießerei 11/1, 69115, Heidelberg, Germany, trial code: S-176/2020) and in compliance with the principles stated in the Declaration of Helsinki and its later amendments. Patient’s written consent was obtained in advance, except in cases of incapacity, in which consent was obtained from legal representatives.

### Data collection and standard laboratory parameters

An assessment of socio-demographic and clinical features was conducted, including pre-existing comorbidities based on the Charlson comorbidity index^24^. Study-related blood samples anticoagulated with lithium heparin were drawn within 24 h after hospitalization and were processed immediately. Following seven days, a second blood sampling was conducted on all patients treated in the intensive care unit (ICU) at the time of study inclusion, if not deceased or transferred to a different hospital. Follow up was performed until death, hospital discharge or 90 days post study inclusion.

### Measurement of sDLL1 and definition of patient cohorts

Quantification of soluble DLL1 (sDLL1) was performed via enzyme linked immunosorbent assay (ELISA) in accordance with manufacturer’s guidelines (Human DLL1 Quantikine ELISA Kit, R&D Technologies, North Kingstown, USA). In brief, plasma derived from untreated, directly centrifuged whole blood samples was used to measure sDLL1 concentration. Based on the previously published studies showing a wide range of different cut off values^11,14,15^ we hypothesized that high levels of DLL1 might show a relationship to clinical outcomes. In this study cohort was divided into two groups: the upper quartile, patients with highest levels of sDLL1 during the study period (`high-sDLL1 group´) and the lower three quartiles of patients with the lowest levels of sDLL1 (`low-sDLL1 group´).

### Outcome variables

In the present study, clinical outcomes of interest comprised the occurrence of secondary infections and the incidence of organ dysfunction as defined below. Further variables included length of ICU and hospital stay as well as 30-day and 90-day mortality.

#### Secondary infections

Secondary infections were defined as the primary detection of infection occurring 48 h after admission to the ICU or normal ward^25^ requiring antimicrobial treatment.

#### Infection site

Secondary respiratory infections were defined as the presence of infectious pathogens in pulmonary specimens, such as sputum or bronchoalveolar lavage fluid, in combination with clinical manifestations of pulmonary involvement, radiographic evidence of pulmonary infiltrates, elevated infection markers such as C-reactive protein (CRP) levels ≥ 10 mg/l or procalcitonin (PCT) levels ≥ 1 ng/ml, and a requirement of antibiotic treatment. Secondary urinary infections were defined as a microbiological identification of pathogens obtained from fluids collected from established urinary catheters, accompanied by elevated infection markers such as C-reactive protein (CRP) levels ≥ 10 mg/l or procalcitonin (PCT) levels ≥ 1 µg/l, as well as the presence of symptoms or clinical signs indicating the need for antibiotic treatment. Secondary catheter infections were defined by the presence of a significant bacterial load in pre-existing catheters, concurrent with acute clinical deterioration, the need for antibiotic therapy, in conjunction with elevated infection markers, including C-reactive protein (CRP) levels ≥ 10 mg/l or ≥ (PCT) levels surpassing 1 µg/l. Secondary bloodstream infections were defined as the detection of infectious pathogens in blood cultures obtained from the patient, in combination with clinical signs of systemic infection, such as fever, chills, or hypotension. The diagnosis was further supported by elevated infection markers, including C-reactive protein (CRP) levels ≥ 10 mg/l or procalcitonin (PCT) levels ≥ 1 µg/l, and a need for antibiotic treatment.

#### Organ dysfunction

Acute pulmonary failure was defined in accordance with the Berlin definition of acute respiratory distress syndrome (ARDS) established by the ARDS Definition Task Force in 2012^26^. This encompasses the presence of acute respiratory distress, characterized by the need for mechanical ventilation. Cardiovascular failure was defined as the need for vasopressors or inotropic support to maintain hemodynamic stability, mean arterial blood pressure > 65 mmHg, and to enhance myocardial contractility to optimize peripheral hypoperfusion and impaired tissue oxygenation, which were measured through serum lactate levels as a marker of increased anaerobic metabolism. Acute renal failure, as per the Kidney Disease: Improving Global Outcomes (KDIGO) criteria^27^, was characterized by a twofold or greater elevation in retention markers along with a deterioration in urinary production or the necessity for dialysis.

### Statistical analysis

For statistical analysis SPSS^®^ Statistics software (Version 29.0.1.0, IBM^®^, Armonk, USA) was used. Data are summarized as median (interquartile range) or mean (SD) in case of continuous variables or absolute number (frequency) in case of categorical variables. Group comparisons were performed using Mann-Whitney U test or Fisher’s exact test as appropriate. We also identified all nosocomial infections and calculated incidence rate as the number of pneumonia cases divided by the number of ICU patient-days, multiplied by 1,000. Results of the latter are reported with odds rations (OR) and 95% confidence intervals (CI). P values < 0.05 were considered statistically significant.

## Electronic supplementary material

Below is the link to the electronic supplementary material.

Supplementary Material 1

## Data Availability

Data is provided within the manuscript or supplementary information files.
