# Supplementary figures and images for: Association of elevated Delta-like canonical Notch ligand 1 levels with clinical outcomes in patients hospitalized for SARS-CoV2 infection
Source: Sci Rep. 2025 May 27;15:18526. doi: 10.1038/s41598-025-03673-6 (PMC12117154; doi:10.1038/s41598-025-03673-6)

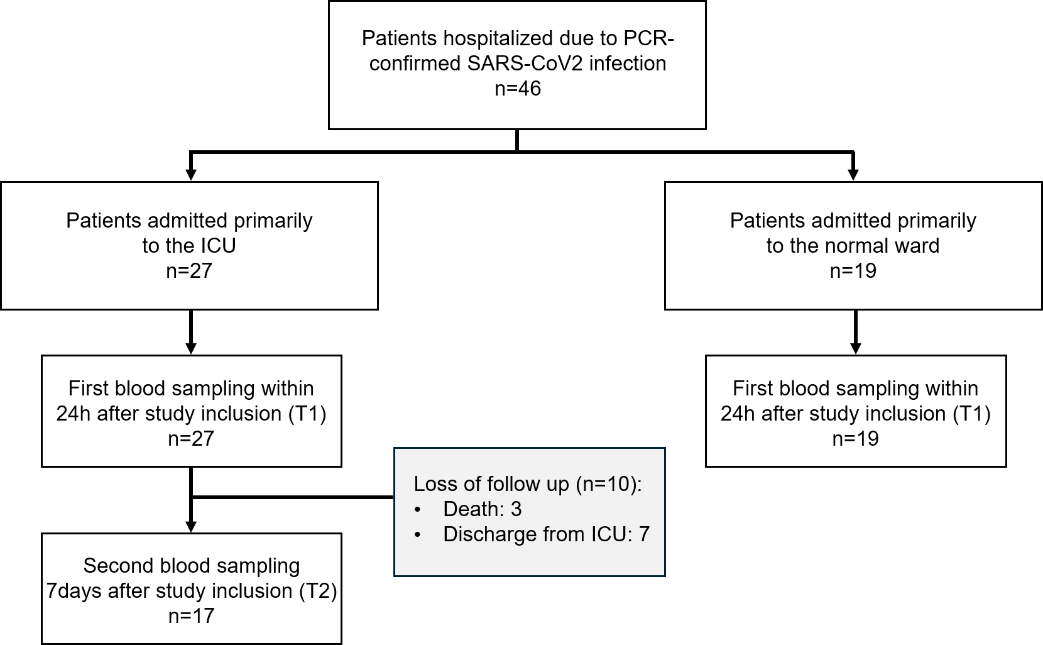


**Supplemental Figure 1. Study cohort.**

Supplement: Supplementary file 1 — Supplementary Material 1 [file 41598_2025_3673_MOESM1_ESM.docx]
